# Case Report: Disseminated hydatid cyst: Unusual presentation and therapeutic challenges

**DOI:** 10.12688/f1000research.142072.2

**Published:** 2024-07-11

**Authors:** Anas Mohamed Babiker, Amro Abdelrahman, Asmaa Abdalkarim, Gufran Algaly, Amin Sanosi, Elhassan Mohamed Abdalla, Hany A Zaki, Moayad Elgassim, Muhnad Abdeen, Mohamed Elgassim

**Affiliations:** 1 Medical Education, Hamad Medical Corporation, Doha, Doha, Qatar

**Keywords:** Disseminated hydatid cyst, Cystic echinococcosis, Spleen cyst, Lung cyst, Liver cyst, Case report

## Abstract

**Introduction:** Cystic echinococcosis (CE), caused by *Echinococcus granulosus sensu lato*, is a parasitic disease prevalent in endemic regions. CE frequently leads to the formation of hydatid cysts in various organs, with the liver being the most commonly affected site. Involvement of the spleen has been rarely reported in the literature. Managing disseminated hydatid cyst disease presents significant diagnostic and therapeutic challenges.

**Case presentation:** A 40-year-old female with a past medical history of hypothyroidism presented with sudden onset shortness of breath, dry cough, and chest pain for 3 days. She had a recent travel history to Egypt. Physical examination revealed mild right upper quadrant tenderness. Laboratory findings showed elevated white blood cell count with eosinophilia and increased inflammatory markers. Chest X-ray and pan-computed tomography (Pan-CT) scans identified multiple cystic lesions in the lung, liver and spleen. Serological tests confirmed the presence of anti-Echinococcus antibodies, leading to a diagnosis of disseminated hydatid cyst disease. The patient was managed medically and surgically by a multidisciplinary team.

**Conclusion:** Disseminated hydatid cyst disease, though rare, presents complex diagnostic and management challenges. Timely recognition, supported by clinical, radiological, and serological assessments, is essential. Surgical intervention should be considered in a patient when multiple extrahepatic cysts are present, and rupture is evident, as this approach can significantly reduce patient morbidity and mitigate life-threatening complications.

## Introduction

The hydatid disease, cystic echinococcosis (CE), is a parasitic disease caused by *Echinococcus granulosus sensu lato.* In endemic countries, CE shows an average incidence of 200 per 100,000 populations with a mortality range of 2–4% when appropriate treatment is provided promptly. ^1^ The larval form of the deadly tapeworm may lodge itself in various different sites and organs to form fluid-filled sacs known as hydatid cysts. The disease may manifest through dysfunction of the organs where the cysts form. With the liver being the most vulnerable, it alone accounts for nearly 70% of cases, while the lungs account for 20% of deposits. Other organs may also be involved in other rarer cases, including the kidneys, muscles, brain, and spleen. ^2^ In this case, we are reporting a 40-year-old female patient that presented to the hospital with sudden onset shortness of breath, a dry cough, and chest pain for only 3 days. Radiological reports showed hydatid cyst involvement of the liver, the lungs, and the spleen, with positive antibodies on serological studies. She was diagnosed as a case of disseminated hydatid cyst and managed by a multidisciplinary team.

## Case presentation

A 40-year-old Egyptian female, previously diagnosed with hypothyroidism and currently taking thyroxine, presented to the hospital with sudden onset shortness of breath, a dry cough, and chest pain for 3 days. The patient denied any orthopnea or paroxysmal nocturnal dyspnea. The review of other systems was unremarkable. Apart from thyroxine, the patient was not taking any other medications. She had traveled to Egypt two months before her presentation. In the emergency department, her vital signs were within normal limits. Her temperature was 36.7°C, her blood pressure was 136/72 mmHg, and she had a heart rate of 86 beats per minute, all while maintaining normal saturation on room air. On physical examination, she was in distress; auscultation of her chest was clear with no crackles or rhonchi. An abdominal examination revealed only minor tenderness in the right upper quadrant. Examination of other systems was unremarkable. Results of her lab investigations revealed a high white blood cell (WBC) count of 12,000 cells/μl (normal range=4,000–11,000 cells/μl) with prominent eosinophils at 3.5 cells/μl (normal range=0–0.5 cells/μl) and elevated C-reactive protein level of 25 mg/L (normal range= 0–5 mg/L). Initial impression was either heptic hydatid cyst or hepatic granuloma.

**Figure 1.**
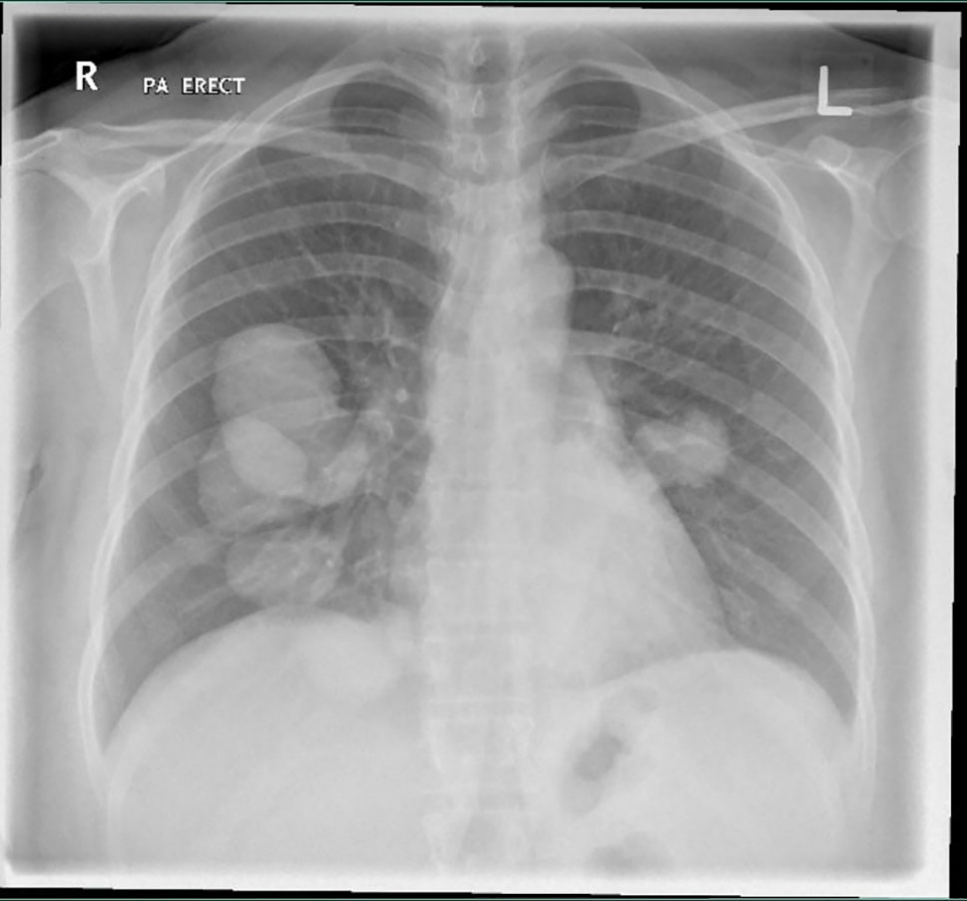
Chest X-ray. Multiple well-defined radiopaque rounded densities (Cannon balls) scattered throughout both lungs.

A chest X-ray showed multiple well-defined radiopaque rounded densities (Cannon balls) scattered throughout both lungs and more on the right side ( Figure 1).

**Figure 2.**
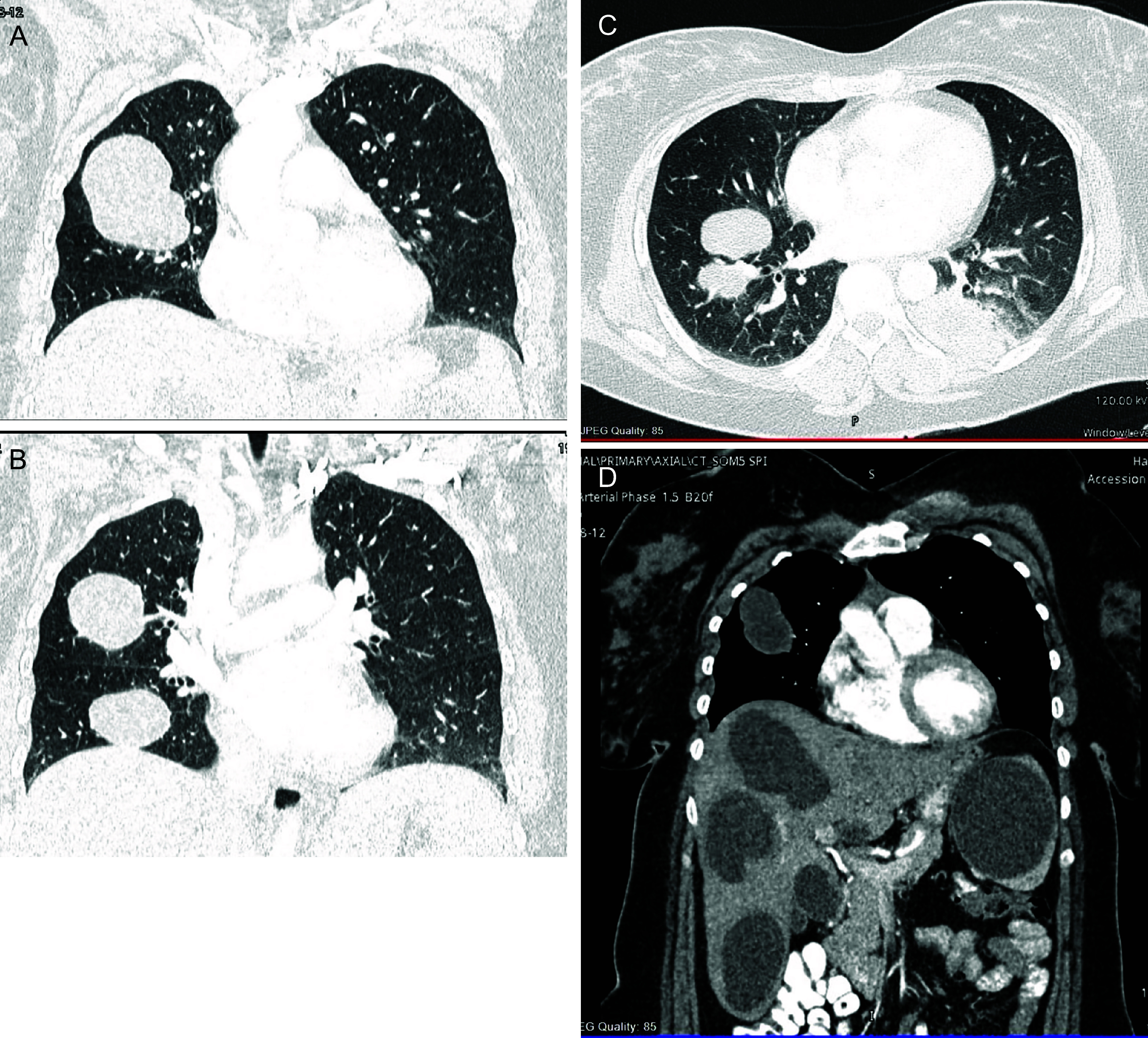
Axial and coronal selected images. (A) Well-circumscribed bilateral pulmonary cystic lesions representing hydatid cysts. The largest cyst measured 6 cm in the anterior segment of the right upper lobe. (B) Bilateral multiple cysts. (C) Ruptured hydatid cyst with surrounding consolidation in the posterior basal region of the left lobe. (D) Multiple enlarged, variable-size subcapsular cystic lesions with multiple curvilinear hyperattenuating structures in both lobes of the liver. The largest is a 5-cm-segment V/VI image with no postcontrast enhancement. A 7.5-cm-enhancing cystic lesion in the upper pole of the spleen.

The radiologist recommended a pan-computed tomography (PAN CT) scan to look for cystic involvement on other organs, which revealed multiple large, well-circumscribed bilateral pulmonary cystic lesions representing hydatid cysts. The largest cyst measured 6 cm in the anterior segment of the right upper lobe. In the posterior basal region of the left lobe, a ruptured hydatid cyst with surrounding consolidation was discovered. Furthermore, multiple enlarged, variable-size subcapsular cystic lesions with multiple curvilinear hyperattenuating structures were seen in both lobes of the liver. The largest is a 5-cm-segment V/VI image with no postcontrast enhancement. A 7.5-cm-enhancing cystic lesion in the upper pole of the spleen was also seen ( Figure 2).

**Figure 3.**
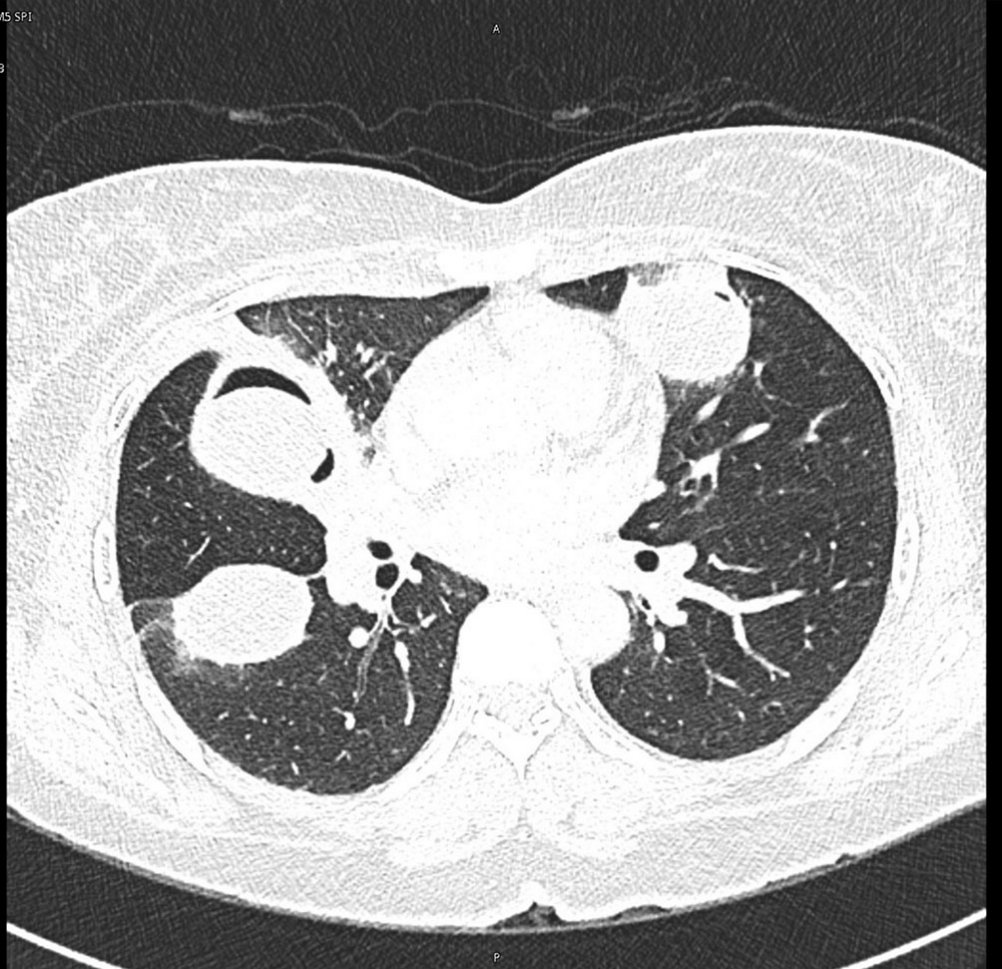
CT scan of the chest. Ruptured cyst in the right upper lobe of the lung.

The image findings supported the diagnosis of disseminated hydatid disease, and serological tests for echinococcus antibodies were positive. Urgent consultations were obtained with both the infectious disease and thoracic surgery teams. The infectious disease (ID) team recommended 400 mg of albendazole orally twice daily with follow-up until resolution. The thoracic surgery team advised her to return to the clinic after two weeks of stabilization for elective surgery and to consult with hepatobiliary surgeons for liver and spleen cysts. The plan was to surgically remove the lung cysts first, followed by the liver and spleen cysts later, along with albendazole, which was prescribed by the ID team. After three days of stabilization and resolution of symptoms, the patient was discharged with an outpatient follow-up with the infectious disease, thoracic surgery, and hepatobiliary surgery teams. One week after being discharged, the patient returned with a severe dry cough and chest pain. A new CT scan of the chest revealed that a new cyst had ruptured in the right upper lobe of the lung ( Figure 3).

**Figure 4.**
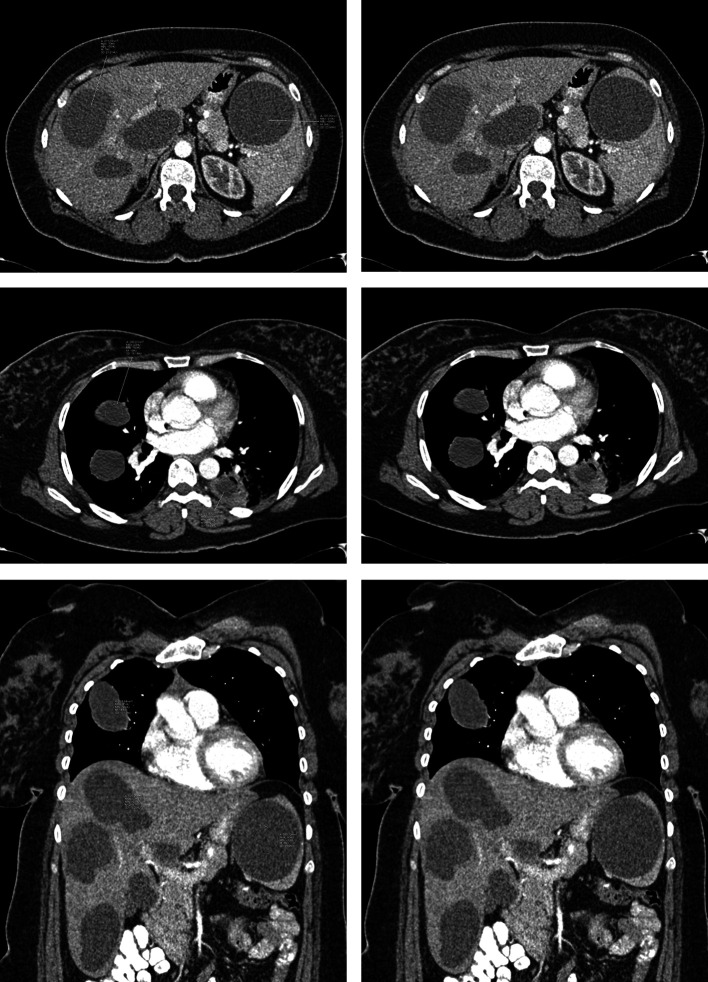
Multiple selected axial and coronal images of the abdomen and chest showing multiple cysts with average Hounsfield unit value of around zero indicating the fluid content.

The patient underwent an emergency thoracotomy with multiple cystectomies, and histopathological examination of the surgical specimen revealed findings consistent with hydatid cysts. Six months later, a follow-up CT scan of the chest showed cyst resolution. The patient had a successful laparoscopic deroofing and endocystectomy of hepatic and splenic cysts one month later, and the pathological analysis of the surgical specimen from hepatic and splenic tissues showed findings consistent with a hydatid cyst.

## Discussion

Hydatid cysts are categorized as zoonotic infections that primarily affect dogs, which serve as definitive hosts when they consume contaminated sheep, goats, or various animal tissues. A single cyst can contain numerous protoscolices, each capable of maturing into an adult tapeworm upon ingestion by the definitive host. Animals acquire this infection through the consumption of contaminated food. In humans, accidental intermediate hosts become infected through the consumption of infected food or water, contact with contaminated soil, or direct contact with definitive hosts. The larval stage enters the bloodstream and infiltrates various organs in the body, particularly those with high blood supplies. ^3^^,^^4^ Echinococcus granulosus cysts can inhabit any anatomical site. The clinical manifestation depends on several factors, including the affected organ, specific location within the organ, stage of cyst development, and vitality of the cyst contents. In most cases, these cysts remain silent and are often discovered incidentally during routine imaging or postmortem examinations. However symptoms may arise as a result of cyst enlargement, causing pressure effect on adjacent structures, potential infection, or the rupture of cyst contents into adjacent body cavities. ^5^ After infection, the parasitic infection can disseminate to different organs via the lymphatic or venous system, with the liver being the most frequently affected organ initially. Appropriate treatment reduces the risk of further spread. ^6^ Our patient presented with a cough, shortness of breath, and chest pain due to a ruptured cyst. In addition to that, his physical examination showed right upper abdominal tenderness.

The diagnosis of a hydatid cyst can be established through combination of clinical assessment, laboratory and serological studies, radiological evidence, and histopathological analysis. In addition to identifying elevated inflammatory markers and a high eosinophil count in the bloodstream, serological tests of our patient also revealed the presence of anti-Echinococcus antibodies. A previous research study has proposed that eosinophilia in individuals afflicted with echinococcosis lacks specificity and has advocated for the inclusion of serological studies in the diagnostic process. These serological tests exhibit a sensitivity of 80% to 100% for hepatic echinococcosis, 50% to 56% for pulmonary echinococcosis, and 25% to 50% for detecting involvement of other organs. ^7^ Generally, the classic radiological finding of a hydatid cyst of the lung is a well-defined oval to rounded opacity ranging from 1 to 20 cm in size. Usually, lung cysts are located in the lower lobes (60% of cases) and can be multiple and bilateral. ^8^ In cases of hepatic involvement, the most common affected portion is the right portion of the liver. The third most common site of involvement, following the liver and the lung, is the spleen. Splenic hydatid cysts are relatively rare and usually solitary, and their imaging characteristics are similar to those of hepatic hydatid cysts. ^9^ A previous study also reported a case of disseminated echinococcosis involving the lung, liver, and spleen with predominantly abdominal symptoms and pleural effusion on radiological studies. ^10^ In our case, a chest X-ray was ordered, and it showed various rounded radiopaque densities, which prompted further evaluation by a pan-computed tomography (pan-CT) scan. The pan-CT results showed multiple cystic lesions in both lungs. The largest cyst was 6 cm in size and located on the left lobe, and a ruptured cyst on the right lobe was also noted. In addition to pulmonary cysts, multiple cystic lesions involving both lobes of the liver and spleen were also detected, supporting the diagnosis of disseminated echinococcosis.

The management of hydatid cysts is both medical and surgical. The indications for surgical intervention are a large cyst size of 10 cm, concomitant extrahepatic involvement, and an infected cyst. ^11^ There are two types of surgical intervention: radical (pericystectomy) and conservative (partial cystectomy); the surgery type is usually determined based on the cyst’s size and the presence of cysts outside the liver. In a systematic review, researchers compared the results of radical and conservative surgery with and without a course of chemotherapy postoperatively. The result of radical surgery followed by a course of antiparasitic medication has shown a better outcome in terms of length of hospital stay and post-surgical complications compared to conservative surgery. However, the time of radical surgery was longer with a larger amount of blood loss, and additionally, use of antiparasitic medication after surgery showed a better outcome with reduced recurrence regardless of the type of surgery. ^12^ Albendazole is the standard medication for hydatid cysts, as recommended by the World Health Organization (WHO). It is prescribed both before and after surgery to reduce cyst size, prevent relapses, sterilize the cyst, and mitigate post-surgical complications. ^13^ Our patient started first on medical treatment and planned for elective surgery by the cardiothoracic team, but because she developed symptoms a week after resuming medication after the rupture of an additional cyst, she underwent an urgent right thoracostomy with cystectomy, followed by histopathological analysis of the surgical specimen, which showed findings suggestive of a hydatid cyst. The patient was discharged on albendazole oral tablets. On her 6-month follow-up, a CT scan of the lungs showed evidence of resolution of the hydatid cyst. One month later, elective laparoscopic deroofing and endocystectomy for hepatic and splenic cysts were done, and the surgical specimen was investigated in the histopathology lab. The results showed findings consistent with a hydatid cyst.

## Conclusion

Prompt suspicion and diagnosis of a ruptured hydatid cyst are vital, particularly in patients with respiratory symptoms and radiographic evidence of cystic lesions, especially in those originating from endemic regions. Early surgical intervention is strongly advised when multiple extrahepatic cysts are present, and rupture is evident, as this approach can significantly reduce patient morbidity and mitigate life-threatening complications.

### Case report consent

Written informed consent for publication of their clinical details and/or clinical images was obtained from the patient.

## Data Availability

All data underlying the results are available as part of the article and no additional source data are required. Zenodo: CARE checklist for “Case report: Disseminated hydatid cyst: Unusal presentation and therapeutic challenges.” https://doi.org/10.5281/zenodo.8398651. ^14^ Data are available under the terms of the Creative Commons Attribution 4.0 International license (CC-BY 4.0).
