# Supplementary material for: Case Report: Disseminated hydatid cyst: Unusual presentation and therapeutic challenges
Source: F1000Res. 2024 Jul 11;12:1492. Originally published 2023 Nov 22. [Version 2] doi: 10.12688/f1000research.142072.2 (PMC11267145; doi:10.12688/f1000research.142072.2)
Supplement: Peer review history

# Reviewer report: Reviewer response for version 2

**DOI:** 10.5256/f1000research.167482.r234526

**Published:** 2024-08-02

**Authors:** Ali Bilal Ulas

**Affiliations:** 1 Ataturk University, Erzurum, Turkey

The authors describe the presentation and management of a 40-year-old female patient with disseminated hydatid cyst disease involving the lungs, liver and spleen. The patient presented with sudden onset of shortness of breath, dry cough and chest pain. The diagnosis was confirmed by clinical, radiological and serological investigations. The patient was treated with a combination of medical and surgical interventions. The report attempts to highlight the complexity and therapeutic challenges associated with disseminated hydatid cyst disease.

 I have a few suggestions that might help to improve the manuscript:

 First, although the case is thoroughly presented, it would be great to see a more in-depth discussion of the diagnostic and treatment decisions made. Providing more insight into why certain diagnostic tools were chosen over others and how these choices affected the patient's outcome could enrich the reader's understanding and highlight the complexities involved. The inclusion of a summary table of key diagnostic findings could also improve clarity.

 The rationale for choosing certain treatment strategies over others could be expanded, and a discussion of the potential risks and complications associated with these treatments is necessary. In addition, the discussion section is brief and does not fully address the implications of the findings or compare them with other cases in the literature. This limits the report's contribution to a broader understanding of the disease. The inclusion of more recent studies and references would ensure a comprehensive and balanced review of the existing literature.

 Although the figures and tables are clear and helpful, their explanatory value could be enhanced by adding more detailed captions. More detailed follow-up of long-term patient outcomes would also add considerable value to the report. Understanding long-term success and any post-treatment challenges would be very informative.

 In addition, highlighting any unique aspects of this case and discussing any potential new findings or lessons learned would be beneficial. Providing a more detailed rationale for the management strategies chosen and discussing alternative approaches would further enhance the manuscript.

 There are a few sentences that could be rephrased for better readability. For instance, some sentences are lengthy and could be broken down into shorter, more digestible parts. For example; Original Sentence: "The patient presented with sudden onset shortness of breath, dry cough, and chest pain, and upon examination, it was found that she had disseminated hydatid cyst disease affecting the lungs, liver, and spleen, which required a combination of medical and surgical interventions." Improved Sentence: "The patient presented with sudden onset shortness of breath, dry cough, and chest pain. Upon examination, she was diagnosed with disseminated hydatid cyst disease affecting the lungs, liver, and spleen. This condition required a combination of medical and surgical interventions." Reducing redundancy can make the manuscript more concise. For example; Original Sentence: "The patient was diagnosed with disseminated hydatid cyst disease affecting multiple organs, including the lungs, liver, and spleen, and the management of the patient involved both medical and surgical treatments to address the condition." Improved Sentence: "The patient was diagnosed with disseminated hydatid cyst disease affecting the lungs, liver, and spleen. Management involved both medical and surgical treatments."

 Although the case is presented with clear and comprehensive clinical details, the need for indexing is questionable for the following reasons:

 Commonplace nature: There are many similar cases in the literature, and large series have detailed the management of disseminated hydatid cyst disease.

 Given these considerations, I respectfully believe that this manuscript does not add sufficiently to the existing literature for indexation at this time..

Are enough details provided of any physical examination and diagnostic tests, treatment given and outcomes?

Yes

Is the case presented with sufficient detail to be useful for other practitioners?

Partly

Is sufficient discussion included of the importance of the findings and their relevance to future understanding of disease processes, diagnosis or treatment?

Partly

Is the background of the case’s history and progression described in sufficient detail?

Partly

Reviewer Expertise:

Thoracic surgery

I confirm that I have read this submission and believe that I have an appropriate level of expertise to state that I do not consider it to be of an acceptable scientific standard, for reasons outlined above.

## References

[1] : Large Case Series Analysis of Cystic Echinococcosis. *Indian Journal of Surgery* .2021;83(S4) : 10.1007/s12262-021-03061-0 897-906 10.1007/s12262-021-03061-0

# Reviewer report: Reviewer response for version 2

**DOI:** 10.5256/f1000research.167482.r301751

**Published:** 2024-07-23

**Authors:** Sultan Abdulwadoud Alshoabi

**Affiliations:** 1 Department of Diagnostic Radiology, College of Applied Medical Sciences, Taibah University, Medina, Al Madinah Province, Saudi Arabia

This case report appears good for indexing.

Are enough details provided of any physical examination and diagnostic tests, treatment given and outcomes?

Partly

Is the case presented with sufficient detail to be useful for other practitioners?

No

Is sufficient discussion included of the importance of the findings and their relevance to future understanding of disease processes, diagnosis or treatment?

Partly

Is the background of the case’s history and progression described in sufficient detail?

Partly

Reviewer Expertise:

Radiology, Medical Imaging

I confirm that I have read this submission and believe that I have an appropriate level of expertise to confirm that it is of an acceptable scientific standard.

# Reviewer report: Reviewer response for version 1

**DOI:** 10.5256/f1000research.155570.r224736

**Published:** 2024-02-14

**Authors:** Khawaja Bilal Waheed

**Affiliations:** 1 King Fahad Military Medical Complex, Dhahran, Saudi Arabia

The authors did not mention why the radiologist suggested a PAN CT. Also, while providing a caption for CT scan images, please correct the caption as it is not PAN CT but rather selected axial, and coronal images in lung and abdominal windows.

 The authors did not provide differential diagnosis for this case, both at initial presentation or in discussion part.

 It would be better to include a histopathological specimen picture or slide demonstrating the hydatid cyst.

Are enough details provided of any physical examination and diagnostic tests, treatment given and outcomes?

Partly

Is the case presented with sufficient detail to be useful for other practitioners?

Yes

Is sufficient discussion included of the importance of the findings and their relevance to future understanding of disease processes, diagnosis or treatment?

Yes

Is the background of the case’s history and progression described in sufficient detail?

Partly

Reviewer Expertise:

Radiology and imaging

I confirm that I have read this submission and believe that I have an appropriate level of expertise to confirm that it is of an acceptable scientific standard, however I have significant reservations, as outlined above.

# Response

**Published:** 2024-03-26

**Authors:** Amro Abdelrahman

**Affiliations:** Hamad Medical Corporation, Doha, Doha, Qatar

Dear reviewer.

 Thanks for feedback.

 We will add that pan ct was recommended to look for other cystic involvemen in othe organs.

 We will correct the figure issue.

 We will add differential diagnosis in case presentation

 Thanks again,

# Reviewer report: Reviewer response for version 1

**DOI:** 10.5256/f1000research.155570.r234518

**Published:** 2024-02-13

**Authors:** Gian Luca D'Alessandro, Pietro Rinaldi

**Affiliations:** 1 Department of Clinical-Surgical, Diagnostic and Pediatric Sciences, University of Pavia, Pavia, Italy; 2 Division of Thoracic Surgery, Azienda Ospedaliera Nazionale Santi Antonio e Biagio e Cesare Arrigo Alessandria, Alessandria, Piedmont, Italy; 3 Department of Molecular Medicine, University of Pavia, Pavia, Lombardia, Italy

In the case reported by A. M. Babiker et al. the patient, an Egyptian woman aged 40 y.o., sought medical attention for respiratory symptoms and multiple parenchymal cystic lesions: 3 in the right lung, 1 in the left lung, at least 3 in the liver and 1 in the spleen. Respiratory symptoms (shortness of breath, cough and chest pain), radiological picture, eosinophilia and positive *E. granulosus* serology suggested the diagnosis of multi-organ CE complicated by rupture of the left lung cyst.

 Although no standardized definition of disseminated hydatidosis exists, in our opinion the term should be limited to secondary echinococcosis, i.e. the dissemination of cyst following spillage and seeding of protoscoleces after leakage or rupture, spontaneous or otherwise typically in the peritoneal or pleural cavities. By this definition, this patient would have multi-organ echinococcosis instead, because the cysts are all intra-parenchymal.

 In lung CE the risk of cyst rupture is higher than in the liver with potential complications such as bronchial and pleural ^1^. The first chest CT of the patient showed the rupture of one lung cyst into the pleural space with pericystic consolidation. Complicated lung hydatid disease can be dangerous, even deadly (anaphylaxis, asphyxia) and, for this reason, early treatment is essential.

 The authors correctly based their hypothesis on clinical picture, imaging findings and serology. However, serology only has a confirmatory role and a negative serology does not rule out echinococcosis. Indeed, CE diagnosis is mainly made through imaging. The positive serology can be explained by the presence of multiple cysts with rupture, factors increasing serology sensitivity in the lung. We are interested in knowing which type of serologic test has been used.

 After diagnosis, the patient was treated with albendazole (ABZ) and the thoracic surgical team suggested to defer surgery to completion of two weeks of medical therapy. Therefore, the woman was discharged and a week later presented again to the E.D. with the rupture of one contralateral lung cyst.

 We argue that the patient should have been operated immediately at first presentation, without delay. Cysto-pleural fistula poses risk of pleural dissemination and disease relapse and would have required immediate thoracoscopic pleural toilette with protoscolicidal agents (hypertonic solution, iodopovidone), under ABZ prophylaxis (starting the day before the procedure). As second step, the intact right lung cysts could be removed (immediately after stabilization, i.e. one week later).

 The use of ABZ in the lung is controversial: in uncomplicated lung CE, starting ABZ is not urgent since the risk of treatment-induced cyst rupture (cyst membrane detachment and opening of cysto-bronchial fistula), while it is indicated as peri-operative prophylaxis ^1^. In the present case, the patient had complicated disease with pleural fistula and required ABZ to prevent the larval spillage (secondary echinococcosis), but in so doing the other lung uncomplicated cysts were damaged by medical therapy and ruptured, requiring emergency surgery. We guess that a close monitoring of the patient without interrupting hospitalization would have been more beneficial for such complications. In the conclusion of their report, the colleagues state that “early surgical intervention is strongly advised when multiple extrahepatic cysts are present and rupture is evident”, but questionably choose to discharge the patient with ABZ. It is likely that the second rupture may have been triggered by medical treatment.

 We agree with the authors in the choice of a sequential surgical approach prioritizing lung cysts: in multi-organ echinococcosis, due to the higher risk of complication of lung disease, thoracic surgery should be scheduled as quickly as possible and before handling abdominal disease. In some instances, i.e. parasitic cysts of the right liver with concomitant supradiaphragmatic lung cysts, same-time surgery (chest and liver) can be performed by experienced surgeons. Otherwise, surgery in two times is an acceptable option, unless in case of complicated abdominal cysts.

 The authors adequately chose a parenchymal-sparing technique, according to expert recommendations ^1^ ^2^. No information is given about the use of surgical field protection (pads soaked with hypertonic saline) and, more importantly, about the management of the left lung cyst complicated by rupture: as highlighted above, pleural fistula requires surgical toilette, otherwise the patient is at high risk of secondary echinococcosis. We are interested in knowing how did the authors deal with the left lung ruptured cyst.

 The authors said that the patient was discharged with oral ABZ after the first surgery for lung disease, but we did not find details about the timing of coverage and if the ABZ prophylaxis was used for the subsequent abdominal surgery: according to recommendations, a 1-3 months course of ABZ after each surgical act is required.

 Some remarks about the Introduction:

- Incidence of CE (number of new infections/year) is difficult to calculate because the disease lacks an acute symptomatic phase after infection and therefore can go unnoticed. The surrogate epidemiological parameter is the number of new diagnosis/year, based on symptomatic cases seeking medical care. Unfortunately, this measure does not reproduce the real burden of disease, because a great majority of CE cases are asymptomatic, hence not reported.
- We suggest to change the reference n.1 in relation with CE epidemiology and mortality rate, since the paper by Schweiger et al. is about alveolar echinococcosis (AE). The topic is well described in the review by Wen et al. ^3^.

In the discussion chapter:

- We would like to comment on the sentence “a single cyst can contain numerous protoscoleces”: strictly speaking, a single hydatid fertile cyst contains hundreds to thousands of protoscoleces, as shown in studies carried out in the intermediate host ^4^.
- A more accurate description of the definitive host infection should be given: canids acquire the metacestode through ingestion of parasitized viscera of the intermediate host.
- Diagnosis of CE is mainly based on imaging methods. Others elements (clinical picture, laboratory examination and serology) are ancillary, but not conclusive without imaging ^5^. Microscopic evidence of protoscoleces in cyst fluid taken with surgery or aspiration allows direct diagnosis, although not always feasible or essential.
- CE is not a frequent cause of eosinophilia, but when eosinophilia is detected in a patient from an endemic area, CE should be considered as eosinophilia suggests leakage or rupture ^6^.

Treatment of CE is complex and indications are different in lung and liver disease. In liver CE and broadly speaking in abdominal CE, surgery is not necessarily the best option and should be based primarily on a stage-specific approach, as per WHO recommendations ^1^:

- CE1 and CE3a liver cysts do not need necessarily need surgery and can be managed with ABZ or with percutaneous treatments, according to their size;
- CE4 and CE5 liver cysts do not need any treatment if uncomplicated. They need only ultrasound follow-up to capture relapses (watch and wait);
- CE2 and CE3b stage are cured by surgery, as they respond poorly to medical or percutaneous treatments other than Modified Catheterization techniques;
- Complicated liver cysts (i.e. biliary fistula, infected cysts) require surgery.

Conversely surgery is the only treatment for lung CE with prompt partial cystectomy as first option ^5^.

 In the colleague’s paper no information about the cyst stage is available and this point is noteworthy and should be addressed because it is crucial for choosing the treatment. Apparently, an ultrasound examination of liver and splenic cysts has not been performed, hence cysts stage is lacking.

 In this case, if abdominal cysts were active cysts (CE1 stage), they could have potentially been treated with medical or percutaneous treatment, turning to surgery once conservative therapy had failed. We read that laparoscopic endocystectomy was performed on liver and splenic cysts. It would be useful to share details of the procedure on the spleen: was spleen removed together with the cyst? Spleen-sparing surgical options need to be considered because splenectomized patients are at significant risk of infection, with overwhelming post-splenectomy sepsis (OPSI) as the most serious complication (vulnerability to invasive infections by encapsulated bacteria: *S. pneumoniae*, *H. influenzae*, *N. meningitidis*). Prevention with vaccination against such pathogens is recommended ^7^.

 Finally, it would be useful to know if clinical and radiological follow-up is planned: if available, we would appreciate to evaluate imaging of lungs and abdomen after surgery.

Are enough details provided of any physical examination and diagnostic tests, treatment given and outcomes?

Yes

Is the case presented with sufficient detail to be useful for other practitioners?

Yes

Is sufficient discussion included of the importance of the findings and their relevance to future understanding of disease processes, diagnosis or treatment?

Partly

Is the background of the case’s history and progression described in sufficient detail?

Partly

Reviewer Expertise:

Infectious and tropical diseases department, with expertise in clinical management of patients affected by cystic echinococcosis (CE).

We confirm that we have read this submission and believe that we have an appropriate level of expertise to confirm that it is of an acceptable scientific standard, however we have significant reservations, as outlined above.

## References

[1] : Pulmonary cystic echinococcosis dust. *Curr Opin Infect Dis* .2023;12(1;36) : 10.1097/QCO.0000000000000962 10.1097/QCO.0000000000000962 PMC1048736237578473

[2] : Expert consensus for the diagnosis and treatment of cystic and alveolar echinococcosis in humans. *Acta Trop* .2010;114(1) : 10.1016/j.actatropica.2009.11.001 1-16 10.1016/j.actatropica.2009.11.001 19931502

[3] : Echinococcosis: Advances in the 21st Century. *Clin Microbiol Rev* .2019;32(2) : 10.1128/CMR.00075-18 10.1128/CMR.00075-18 30760475 PMC6431127

[4] : Modelling the age variation of larval protoscoleces of Echinococcus granulosus in sheep. *Int J Parasitol* .2009;39(9) : 10.1016/j.ijpara.2009.01.004 1031-5 10.1016/j.ijpara.2009.01.004 19504758

[5] : Cystic Echinococcosis. *J Clin Microbiol* .2016;54(3) : 10.1128/JCM.02420-15 518-23 10.1128/JCM.02420-15 26677245 PMC4767951

[6] : Eosinophilia and cystic echinococcosis: what is the relationship?. *Trans R Soc Trop Med Hyg* .2020;114(1) : 10.1093/trstmh/trz105 16-22 10.1093/trstmh/trz105 31728535

[7] : Recommended vaccinations for asplenic and hyposplenic adult patients. *Hum Vaccin Immunother* .2017;13(2) : 10.1080/21645515.2017.1264797 359-368 10.1080/21645515.2017.1264797 27929751 PMC5328222

# Response

**Published:** 2024-03-26

**Authors:** Amro Abdelrahman

**Affiliations:** Hamad Medical Corporation, Doha, Doha, Qatar

Dear reviewer,

 Thanks for feedback. 

 It would be more practical if you rewrite your points in a bullet point writing style.

# Reviewer report: Reviewer response for version 1

**DOI:** 10.5256/f1000research.155570.r234514

**Published:** 2024-02-13

**Authors:** Sultan Abdulwadoud Alshoabi

**Affiliations:** 1 Department of Diagnostic Radiology, College of Applied Medical Sciences, Taibah University, Medina, Al Madinah Province, Saudi Arabia

1.  At the end of the introduction, the authors should write the purpose of this case report and should explain the importance, what is new in this case, and what they want to emphasize by this case report.
2. In case presentation, the authors should write the events as a scenario by periods of time. They should give more details about radiological examinations because radiological examinations are the the clue for diagnosis of hydatid cysts, and usually pathognomonic for hydatid cyst. The authors should add CT image of chest with mediastinal window to show the contents of the fluid cysts. It is better to add abdominal ultrasound images for liver and splenic cysts if available. They should give wider area for the radiological methods of diagnosis of hydatid cyst.
3. In discussion, the authors should compare their findings with previous similar results either in radiological findings or in surgical intervention and outcomes.
4. Other minor corrections such as adding units of WBCs and others are needed.

Are enough details provided of any physical examination and diagnostic tests, treatment given and outcomes?

Partly

Is the case presented with sufficient detail to be useful for other practitioners?

No

Is sufficient discussion included of the importance of the findings and their relevance to future understanding of disease processes, diagnosis or treatment?

Partly

Is the background of the case’s history and progression described in sufficient detail?

Partly

Reviewer Expertise:

Radiology, Medical Imaging

I confirm that I have read this submission and believe that I have an appropriate level of expertise to confirm that it is of an acceptable scientific standard, however I have significant reservations, as outlined above.

## References

[1] : Hydatid Disease: A Radiological Pictorial Review of a Great Neoplasms Mimicker. *Diagnostics (Basel)* .2023;13(6) : 10.3390/diagnostics13061127 10.3390/diagnostics13061127 36980435 PMC10047450

# Response

**Published:** 2024-03-26

**Authors:** Amro Abdelrahman

**Affiliations:** Hamad Medical Corporation, Doha, Doha, Qatar

Dear reviewer,

 Thanks for feedback.

 At the end of the introduction, the authors should write the purpose of this case report and should explain the importance, what is new in this case, and what they want to emphasize by this case report.

**We mentioned this in the end of introduction to emphasize on hydatid cyst involvement in liver, lung and spleen and the importance of multidisciplinary team**

**"In this case, we are reporting a 40-year-old female patient that presented to the hospital with sudden onset shortness of breath, a dry cough, and chest pain for only 3 days. Radiological reports showed hydatid cyst involvement of the liver, the lungs, and the spleen, with positive antibodies on serological studies. She was diagnosed as a case of disseminated hydatid cyst and managed by a multidisciplinary team."**

 In case presentation, the authors should write the events as a scenario by periods of time. They should give more details about radiological examinations because radiological examinations are the the clue for diagnosis of hydatid cysts, and usually pathognomonic for hydatid cyst. The authors should add CT image of chest with mediastinal window to show the contents of the fluid cysts. It is better to add abdominal ultrasound images for liver and splenic cysts if available. They should give wider area for the radiological methods of diagnosis of hydatid cyst.

**We think our case was written as periodic events including initial presentation at ED, 1 week post discharge presentation and 6-month follow up post surgery.**

**We will add mediastinal image to emphasize on radiological assessment.**

 In discussion, the authors should compare their findings with previous similar results either in radiological findings or in surgical intervention and outcomes.

**We made comparison just in a different way. In 1st and 2nd paragraph we present findings of literature and at the end of the paragraphs we present our findings.**

 Other minor corrections such as adding units of WBCs and others are needed.

**Units will be added.**
